# Functionality of Djulis (*Chenopodium formosanum*) By-Products and In Vivo Anti-Diabetes Effect in Type 2 Diabetes Mellitus Patients

**DOI:** 10.3390/biology10020160

**Published:** 2021-02-17

**Authors:** Po-Hsien Li, Yung-Jia Chan, Ya-Wen Hou, Wen-Chien Lu, Wen-Hui Chen, Jie-Yun Tseng, Amanda Tresiliana Mulio

**Affiliations:** 1 Department of Medicinal Botanical and Health Applications, Da-Yeh University, No. 168, University Rd, Dacun, Changhua 51591, Taiwan; 704874@cch.org.tw (W.-H.C.); f338879@ncku.edu.tw (J.-Y.T.); f0665601@cloud.dyu.edu.tw (A.T.M.); 2 College of Biotechnology and Bioresources, Da-Yeh University, No. 168, University Rd, Dacun, Chang-Hua 51591, Taiwan; d0867601@cloud.dyu.edu.tw; 3 Fisheries Research Institute, Council of Agriculture, No. 199, Hou-lh Road, Keelung 202008, Taiwan; ywhou@mail.tfrin.gov.tw; 4 Department of Food and Beverage Management, Chung-Jen Junior College of Nursing, Health Sciences and Management, No. 217, Hung-Mao-Pi, Chia-Yi City 60077, Taiwan; 5 Nutrition Division, Changhua Lukang Christian Hospital, No. 480, Zhongzheng Rd, Lukang, Changhua 50544, Taiwan

**Keywords:** djulis (*Chenopodium formosanum* Koidz.), diabetes mellitus, dietary fibre, physicochemical properties, postprandial glucose

## Abstract

**Simple Summary:**

According to a report from International Diabetes Federation, in 2020 approximately 463 million adults (20–79 years) were living with diabetes, the principles of medical nutrition therapy are to decrease the risk of diabetes by encouraging healthy food choices and physical activity. Djulis is a unique traditional pseudo-cereal crop native to Taiwan. The hull of djulis, which is usually considered to be agricultural waste, is disposed of in landfills and causes some environmental problems. In recent years, many studies have investigated the functional properties of djulis hull. The focus has been on the byproducts of djulis, a waste utilization approach, to further develop enriched functional foods. Djulis hull contained dietary fibre 75.21 ± 0.17% dry weight, and insoluble dietary fibre (IDF) reached 71.54 ± 0.27% dry weight. The IDF postponed the adsorption of glucose and reduced the activity of α-amylase. We found that it is a good source of valuable ingredients that contain a high amount of dietary fibre. Furthermore, for patients with T2DM, consuming djulis hull 30 and 60 min before a meal significantly reduced blood glucose content as compared with patients at the same postprandial times who did not consume it.

**Abstract:**

Djulis (*Chenopodium formosanum* Koidz.) is a species of cereal grain native to Taiwan. It is rich in dietary fibre and antioxidants and therefore reputed to relieve constipation, suppress inflammation, and lower blood glucose. The aim of this study was to investigate the composition and physicochemical properties of dietary fibre from djulis hull. Meanwhile, determination of the in vivo antidiabetic effect on patients with type 2 diabetes mellitus (T2DM) after consuming the djulis hull powder. Djulis hull contained dietary fibre 75.21 ± 0.17% dry weight, and insoluble dietary fibre (IDF) reached 71.54 ± 0.27% dry weight. The IDF postponed the adsorption of glucose and reduced the activity of α-amylase. Postprandial blood glucose levels in patients with T2DM showed three different tendencies. First, the area under the glucose curve was significantly lower after ingesting 10 or 5 g djulis hull powder, which then postponed the adsorption of glucose, but the area under the glucose curve was similar with the two doses. After consuming 10 g djulis hull before 75 g glucose 30 and 60 min after the meal, patients with T2DM had blood glucose values that were significantly lower at the same postprandial times than those of patients who did not consume djulis hull. In short, patients who consumed djulis hull prior to glucose administration had decreased blood glucose level compared with those who did not. Djulis hull may have benefits for patients with T2DM.

## 1. Introduction

Recently, the incidence and prevalence of diabetes has increased rapidly. According to a report from International Diabetes Federation, in 2020 approximately 463 million adults (20–79 years) were living with diabetes; by 2045, this number will increase to 700 million [1]. Furthermore, 374 million people are at increased risk of type 2 diabetes mellitus (T2DM). Diabetes is linked to many chronic complications, especially cardiovascular disease, cerebrovascular disease, kidney disease, neuropathy, and retinopathy [2,3,4]. Diabetes causes a huge expenditure of medical resources, a minimum of US$760 billion in 2019. Hence, early diagnosis of diabetes and control of blood glucose levels is important for preventing and treating diabetes.

Medical nutrition therapy is part of the prevention and treatment of diabetes. The principles of medical nutrition therapy are to decrease the risk of diabetes by encouraging healthy food choices (modifying nutrient intake and lifestyle) and physical activity [5]. A report by the World Health Organization recommended that the intake of dietary fibre in both men and women be >25 g every day. Consuming an adequate amount of dietary fibre may help reduce the prevalence of cancer such as colorectal cancer and ovarian cancer, reduce second-phase glucose-induced hyperinsulinemia after a hypocaloric diet, and maintain the health of the gastrointestinal tract [6,7,8,9]. Moreover, high dietary fibre helps decrease the occurrence of T2DM [10,11].

*Chenopodium formosanum* Koidz. (djulis) is a unique traditional pseudo-cereal crop native to Taiwan that has been used by the aboriginal people for more than 100 years [12]. Djulis is commonly used as fermentation materials for making liquor and as an ingredient in bakery products, snack foods or starchy desserts. Djulis with its colourful appearance has recently garnered enormous attention. The pigment compounds betanin and betacyanin have been studied for their stability and antioxidant capacity [13,14]. In addition, previous studies had clarified that water extracts of djulis effectively protect the liver against carbon tetrachloride-induced liver hepatoxicity and genotoxicity and tert-butyl hydroperoxide-induced oxidative stress in Hep-G2 cells and protected the skin against ultraviolet B induced skin damage [15,16,17].

Djulis contains a high amount of dietary fibre, total phenolics, and total flavonoids which plays a vital role in nutraceutical activity by regulating the antioxidative and apoptotic pathway [18]. The functional components of djulis mainly are phenolic acid and flavonoids such as quercetin, rutin, and chlorogenic acid [19,20,21]. These functional components exhibit strong antioxidant power and also are thought to have anti-inflammatory functions that prevent arteriosclerosis, thrombosis, and carcinogenesis [22,23,24]. However, the study of the dietary fibre properties of djulis and its effect on regulating blood glucose level is limited. 

Byproducts of the food and agricultural industries, such as hulls and shells of nuts and cereals, citrus peels and seeds, and fish viscera extracts have been found to have additional uses, thereby solving waste problems, reducing environmental impacts, and increasing their commercial value [25]. Hence, the focus has been on the byproducts of djulis, a waste utilization approach, to further develop enriched, functional foods. 

The objective of this study was to determine the physicochemical properties of the fibre in djulis hull. We also evaluated the effect of consuming djulis hull powder on blood glucose and insulin contents in patients with T2DM. This study is divided into two parts: (1) analysis of the physicochemical properties of dietary fibre from djulis hull and in vitro experiments to investigate the effects of fibre on glucose diffusion and α-amylase activity; and (2) in vivo analysis of the effect of djulis hull powder on postprandial blood glucose concentrations in patients with T2DM.

## 2. Experimental

### 2.1. Materials

*C. formosanum* was obtained from a local farm at Pingtung, Taiwan. The outer hull that is red coloured (djulis hull) was the research material. Samples were oven-dried before further experimental analysis. After drying, the djulis was crushed by a specially designed shell kernel separator, then sieved to separate the ovary from the outer hull. The djulis ovary and outer hull were collected separately and dried in an oven at 40 °C. After drying, the hull was ground into powder and put through a 100-mesh sieve. The djulis hull powder was stored in hermetically sealed plastic zipper bags and kept in a dry box.

### 2.2. Dietary Fibre

The insoluble dietary fibre (IDF) and soluble dietary fibre (SDF) of djulis were analysed by using the Megazyme kit (Megazyme K-TDFR, Wicklow, Ireland) according to the method of the Association of Official Analytical Chemists (32.1.17). A 1 g of djulis hull powder was added to a 250 mL beaker with 40 mL of 0.05 M MES/TRIS buffer (pH 8.2) and mixed thoroughly. Then 50 mL heat-stable α-amylase solution was added, and the beaker was covered with aluminum foil, placed in a 95 °C water bath for 30 min, removed, and cooled. Then 100 μL of the protease solution was added, followed by placement in a 60 °C water bath for 30 min and stirred occasionally. After cooling, the pH was adjusted to 4.1–4.8 with 0.5 N hydrochloric acid. Next, 200 μL amyloglucosidase solution was added, and the solution was placed in a 60 °C water bath for 30 min. After cooling, the sample solution was transferred to a 50 mL centrifuge tube, with 20 min of 4025× *g* centrifugation. The precipitate (pellet) was removed and washed with 95% ethanol and acetone and then dried in an oven at 70 °C. The weight (Wt1) after drying was the water-IDF. After the supernatant volume was measured, 95% ethanol was added four times, followed by centrifugation at 6511× *g* for 10 min. The precipitate was then washed with 95% ethanol and acetone, dried in an oven at 70 °C, and weighed (Wt2), the water-SDF. The content of water-IDF and water-SDF was corrected by protein and ash content and calculated by Equations (1) and (2)

IDF (%) = [(Wt1 − P − A − B)] / Ws × 100(1)

SDF (%) = [(Wt2 − P − A − B)] / Ws × 100(2)

where Wt1, Wt2 = weight of residue (g); P = protein content of residue (g); A = ash content of residue (g); B = weight of blank (g); Ws = weight of the sample (g).

The water-IDF can be classified into alcohol insoluble solids (AISs) and water-insoluble solids (WISs) depending on the extraction method. The method of quantitative determination of AISs was according to Massiot and Renard [26]. A 20 g amount of djulis hull powder was added to 95% ethanol (1:30, *w/v*), homogenized for 1 min by a homogenizer, then placed into a 75 °C water bath. After stirring for 40 min, the resulting suspension was extracted with a Buchner funnel. The residue was washed with 600 mL of 70% ethanol until the mash, a sugar-free residue, became colourless and was washed twice with 200 mL of 95% ethanol and 15 mL acetone. The residue was dried in an oven at 30 °C and weighed (Wt) as an alcohol-insoluble solid. The content of the alcohol-insoluble solids was corrected by subtracting the protein and ash content and was calculated by Equation (3)

AISs (%) = [(Wt − P − A − B)] / Ws × 100(3)

where Wt = weight of residue (g); P = protein content of residue (g); A = ash content of residue (g); B = weight of blank (g); Ws = weight of the sample (g).

Quantitative analysis of the water-insoluble solids (WISs) in djulis hull referred to the method of Massiot and Renard [26]. A 20 g amount of djulis hull powder was added to the distilled water with 10 times the weight of djulis hull powder (1:10, w/v), homogenized for 1 min at high speed by a homogenizer, followed by centrifugation at 6511× *g* for 20 min. The precipitate was washed with 1200 mL of 70% ethanol until the mash became colourless, a sugar-free residue. Then, it was washed twice with a small amount of 95% ethanol and 15 mL acetone. The residue was dried in an oven at 30 °C and the water-insoluble solid was weighed (Wt. The content of the water-insoluble solids was corrected by subtracting the protein and ash content and calculated by Equation (4):

WISs (%) = [(Wt − P − A − B)] / Ws × 100(4)

where Wt = weight of residue (g); P = protein content of residue (g); A = Ash content of residue (g); B = the weight of blank (g); Ws = the weight of the sample (g).

### 2.3. Determination of Physical and Chemical Properties of Fibres

#### 2.3.1. Bulk Density

By referring to the method of Chau [27] with modifications, a 1 g amount of djulis hull powder was placed in a 10 mL measuring cylinder, and the bottom of the measuring cylinder was tapped on the bench top until the height of the sample could no longer be reduced. The bulk density was calculated by Equation (5)

Bulk density (g/mL) = weight of sample / unit volume of sample(5)

#### 2.3.2. Water-Holding Capacity

Referring to the method of Chau et al. [28] with slight modification, a 0.2 g amount of djulis hull powder was weighed in a 15 mL centrifuge tube, and 10 mL distilled water was added. The sample solutions were left to stand for 24 h, followed by centrifugation for 20 min at 4025× *g*. The supernatant was removed and poured into the measuring cylinder to measure the volume (V). The water-holding capacity was expressed as the volume of water retained per 1 g sample and calculated as in formula (6)

Water-holding capacity (mL/g) = (10 mL − V) / Wt(6)

where V = volume of the supernatant (mL); Wt = weight of sample (g).

#### 2.3.3. Oil-Holding Capacity

Referring to the method of Chau et al. [28] with slight modification, 0.2 g amount of djulis hull powder was weighed in a 15 mL centrifuge tube, and 10 mL sunflower oil was added. The sample solutions were left to stand for 30 min, then centrifuged for 20 min at 1006× *g*. The supernatant was removed and poured into the measuring cylinder to measure the volume (V). The oil-holding capacity was expressed as the volume of oil retained per 1 g of sample and calculated as in formula (7)

Oil-holding capacity (g/g) = (10 mL − V) × 0.88/Wt(7)

where V = volume of the supernatant (mL); Wt = weight of sample (g).

#### 2.3.4. Swelling Properties

Following the method of Ralet et al. [29] with modifications, a 0.2 g amount of djulis hull powder was weighed in a 10 mL measuring cylinder, and 10 mL distilled water was added. The sample solutions were left to stand for 24 h at room temperature. While achieving hydration balances, the volume on the measuring cylinder was recorded. The swelling properties were calculated as in formula (8)

Swelling property = V/Wt(8)

where V = expansion volume of the sample (mL); Wt = initial weigh of the sample (g).

### 2.4. In Vitro Study of the Effect of Djulis Hull Fibre on Glucose Diffusion Rate

#### 2.4.1. Effect of Djulis Hull Fibre on Glucose Diffusion Rate in Glucose–Fibre System

By modifying the method of Ou et al. [30], the glucose–fibre system consists of 50 mmol/L glucose and 0.5 g fibre samples; the control sample had no djulis hull fibre. A dialysis membrane (MW12,000–15,000, size 20, Wako) with a length of 10 cm was prepared by adding 25 mL glucose–fibre system solution. Dialysis was carried out with 150 mL distilled water at 37 °C. After 20, 30, 60, 120 and 180 min, a portion of the dialysate was taken out to determine the glucose content.

#### 2.4.2. Effect of Fibre on Glucose Diffusion Rate in Starch- α-Amylase–Fibre System

Following the method of Ou et al. [30] with modifications, a 10 g amount of potato starch (S-2630, Sigma) was dissolved in 200 mL of 0.05 M phosphate buffer solution (pH 6.5), heated and stirred at 65 °C for 30 min, then cooled to a volume of 250 mL giving a concentration of 4%. The starch- α-amylase-fibre system contains a starch solution, 0.4% (*w/v*) α-amylase (100447, ICN) and fibre samples. A dialysis membrane with length 10 cm (MW 12,000–15,000, size 20, Wako) was prepared by adding a 0.25 g fibre sample, 10 mL starch solution, and 0.04 g α-amylase into the dialysis membrane. Dialysis was carried out with 200 mL distilled water at 37 °C. After 20, 30, 60, 120 and 180 min, a portion of the dialysate was taken out to determine the glucose content. 

#### 2.4.3. Effect of Fibre on α-Amylase Activity

A 0.25 g amount of djulis hull fibre sample was placed in a 50 mL centrifuge tube; then 10 mL potato starch solution and 0.001 g α-amylase were added with vigorous stirring at 37 °C for 1 h. The activity of α-amylase was terminated by the addition of 20 mL of 0.1 N sodium hydroxide solution, with centrifugation at 4025× *g* for 2 min. The content of glucose in the mixture was measured [30].

### 2.5. In Vivo Study of the Effect of Djulis Hull Powder on Postprandial Blood Glucose Content in Patients with T2DM

Patients with T2DM were selected from Department of Endocrinology and Metabolism, Changhua Lukang Christian Hospital, to investigate the effect of djulis hull powder on postprandial blood glucose concentration. Djulis hull powder was used as a dietary fibre supplement to investigate the effect of djulis hull on the blood glucose content in patients after meals. To achieve the effect of ameliorating the blood glucose level, daily intake of fibre should be >50 g. To detect 14.5 to 17.2 g dietary fibre in the daily diet for adults >19 years old, 30 to 35 g dietary fibre supplements daily is required. If divided into three daily meals (breakfast, lunch, and dinner), 10 g of dietary fibre needs to be consumed at each meal. Therefore, this study was designed for consumption of 10 and 5 g djulis hull before a meal to observe its effect on postprandial blood glucose. The djulis hull powder supplements were prepared with 10 g djulis hull powder diluted with 100 mL drinking water and 5 g djulis hull powder diluted with 50 mL drinking water.

We included 12 patients with T2DM, age 30 to 80 years, in this study. Patients could withdraw from the study at any time. We analysed the effect of consumption of only one meal with djulis hull powder on blood glucose within 3 h after the meal. In addition, to control the content of the meal, the tested meal was supplemented with 75 g glucose powder, and the glucose level of the patients was tested by the Oral Glucose Tolerance Test (OGTT) test.

**Figure 1:**
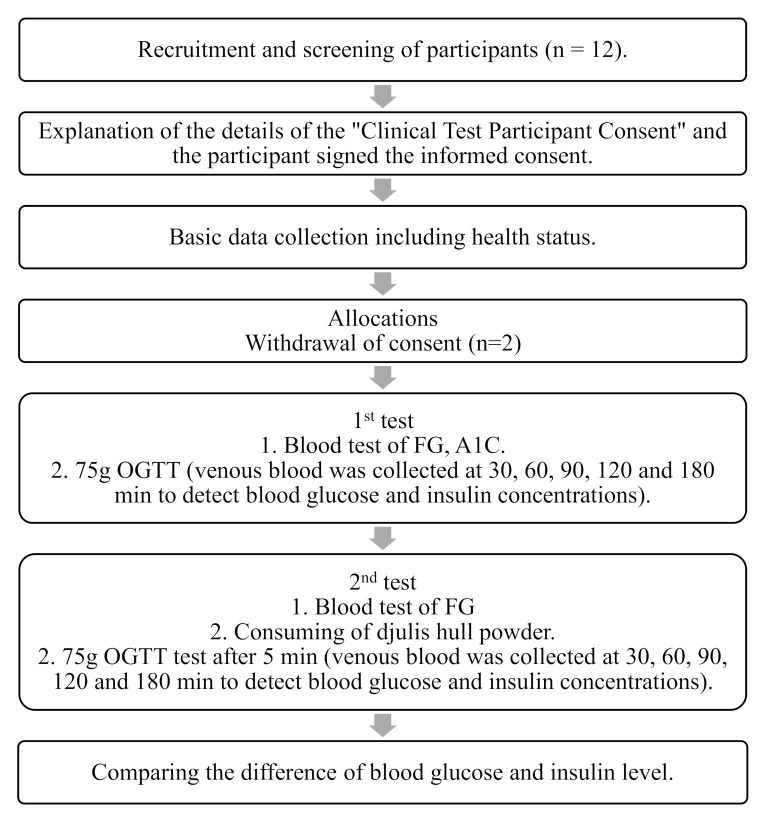
Study protocol of in vivo study the effect of djulis hull powder on the postprandial blood glucose content in patients with type 2 diabetes patients. OGTT, Oral Glucose Tolerance Test.

The overall protocol for the in vivo study of the effect of djulis hull powder on postprandial blood glucose content in patients with T2DM is in Figure 1. First, the 3 h OGTT test was carried out during the first test. The amount of carbohydrate intake by participants the day before the experiment was not <150 g. After fasting overnight for 8 to 12 h, blood glucose was measured in the morning, after which the patients drank 75 g glucose in 250 to 300 mL water within 5 min. The time was counted from the beginning of the drinking the glucose water, and venous blood samples were taken at 30, 60, 90, 120, and 180 min to measure blood glucose. For the second test, after fasting for 8 to 12 h, blood glucose was measured, followed by consumption of the djulis hull powder supplement, and drinking 75 g glucose in 250 to 300 mL water within 5 min. The time was counted from the beginning of the drinking the glucose water, and venous blood samples were taken at 30, 60, 90, 120, and 180 min to measure blood glucose.

#### Ethical Approval and Informed Consent

The study protocol followed Good Clinical Practices and the Declaration of Helsinki and agreed with applicable institutional review board (IRB) regulations. The protocol of the experiment was registered at the Changhua Lukang Christian Hospital (approval no. 102-CCH-IRP-101). The protocol was also approved by the IRB of Changhua Christian Hospital, Changhua, Taiwan (clinical trials approval certificate no. 131003). The participants gave informed consent before beginning any study-related procedures and medications.

### 2.6. Statistical Analysis

Data are presented as mean ± SD. Statistical significance was determined by paired Student *t*-test, by using SPSS 15.0. *p* < 0.05 was defined as statistically significant. The Wilcoxon Signed Ranks Test was used to compare the difference in blood glucose and insulin content between the participants with or without consumption of djulis hull powder. Generalized estimating equations (GEE) methods were used for linear regression analysis to screen the factors affecting postprandial blood glucose and insulin contents.

## 3. Results and Discussion

### 3.1. Analysis of Physical and Chemical Properties of Djulis Hull

#### 3.1.1. Composition Analysis

Different types of dietary fibre content of djulis hull are in Table 1. The total dietary fibre (TDF) content was 75.21 ± 0.17%. The IDF of djulis hull accounted for 71.54 ± 0.27%, whereas the SDF was 3.72 ± 0.45%. The result clarified that the dietary fibre of djulis hull is mainly IDF, consisting of 95.1% of TDF. The water-insoluble solids are classified into AISs and WISs according to the extraction method. The fibre content of djulis hull obtained by different treatment methods is extremely high. The AISs and WISs content of djulis hull was 84.27 ± 0.67% and 81.17 ± 0.33%, respectively. Previous scientific study indicated that the dietary fibre content from *Chenopodium* spp. (*Chenopodium quinoa*), including the TDF, IDF, and SDF, was comparable to the fibre content in this study [31,32]. The IDF from *Chenopodium spp.* was primarily composed of galacturonic acid, arabinose, galactose, xylose and glucose, which play a vital role in controlling obesity, cardiovascular disease, and T2DM [32,33].

#### 3.1.2. Physicochemical Properties of Fibre

The results in Table 2 demonstrate the bulk density, water-holding capacity, swelling property, and oil-holding capacity of the types of dietary fibre in djulis hull. The bulk density of the IDF, AISs and WISs in djulis hull was 0.33 ± 0.11, 0.54 ± 0.14 and 0.37 ± 0.09 g/mL, respectively, which was higher than that of cellulose, 0.29 ± 0.05 g/mL. Djulis hull, because of its higher oil-holding capacity, can be applied in food formulations to stabilize food rich in oil content. In terms of water-holding capacity, the IDF, AISs and WISs contents in djulis hull were 5.27 ± 0.13, 4.02 ± 0.17, and 4.52 ± 0.24 mL/g, respectively, which was also higher than that of cellulose, 3.01 ± 0.19 mL/g. The water-holding capacity found in this study indicates that djulis hull may be used as a functional ingredient to reduce calories, prevent syneresis, and modify the viscosity and texture of some formulated food [34]. The IDF of djulis hull, 7.78 ± 0.24 mL/g, was the highest for swelling property, which was higher than cellulose (7.01 ± 0.22 mL/g), WISs (6.69 ± 0.17 mL/g), and AISs (5.72 ± 0.31 mL/g). The connection between the water-holding capacity and swelling property of fibres was affected by the interaction between the fibre and water, which is because of water surface tension on the capillary structure of fibres. Because of the high water-holding capacity and swelling properties of djulis hull as compared with cellulose, consuming djulis hull can lead to a feeling of fullness and bloating. Hence, for the following clinical tests, the dose of djulis hull used was 10 and 5 g.

### 3.2. In Vitro Determination of the Effect of Fibre on Glucose Diffusion Rate

#### 3.2.1. Effect of Fibre on Glucose Diffusion Rate

The results in Table 3 present the effect of the types of dietary fibre in djulis hull on glucose diffusion rate. Glucose content in dialysate varied by type of dietary fibre in djulis hull as compared with the control group and the cellulose group at 20, 30, 60, 120, 180 min even though the glucose content in dialysate increased with time. The results clarified that the insoluble dietary fibre has a retarding effect on glucose diffusion. In short, the glucose diffusion of all samples was directly proportional to time, and the diffusion rate was significantly lower in the system containing various samples as compared with the control. Therefore, the fibre may have the ability to adsorb glucose. Fibres with dense pores allowing the glucose solution to stay on the surface of the fibre for a longer period can delay the diffusion of glucose. Previous scientific studies with diets containing foods supplemented with oat and barley (dietary fibre sources) β-glucans revealed a reduced glycemic index (GI) and insulinemic response (GII) because the β-glucan decreasing the postprandial glucose response results from high viscosity in the gastrointestinal tract and also decreased starch digestion by α-amylase [35].

#### 3.2.2. Effect of Fibre on α-Amylase Activity

The results in Table 4 show the effect of insoluble dietary fibre from djulis hull on α-amylase activity. Djulis hull insoluble fibre has a direct effect on α-amylase activity, which can hold up or delay the hydrolysis of α-amylase on starch. The AISs and WISs have a significant effect as compared with cellulose and the IDF. The effect of cellulose and the IDF on α-amylase activity did not differ. Overall, the insoluble fibre of djulis hull released was lower than the control group, 26.4% to 76.6% of the control value. The effect of fibre on the activity of α-amylase may be due to the direct adsorption of enzymes and affect the activity. In addition, changes in viscosity, pH, or inhibitors in impure fibres may be responsible for the activity of enzymes [36]. Some pomace or vegetable residue may be physically disturbed by fibre, which delays the action of starch and α-amylase. Some fibre components such as gelatin and polyphenols can also hinder the interaction between starch and enzymes [30].

### 3.3. Effect of Djulis Hull on Postprandial Blood Glucose Concentration in Patients with T2DM

#### 3.3.1. Basic Information of Patients with T2DM

We investigated patients with T2DM with mean diabetes mellitus (DM) duration 4.4 ± 4.0 years, mean age 56.3 ± 13.3 years, mean body mass index 26.8 ± 2.8 kg/m^2^, A1C 6.6 ± 0.9%, body fat rate for males 27.0 ± 3.3% and females 28.1 ± 7.2%, and waist–hip circumference 1.0 ± 0.03 for men and 0.92 ± 0.03 for women. Patients with 10 and 5 g dose of djulis hull did not differ in personal or DM characteristics (Table 5).

#### 3.3.2. Comparison of Blood Glucose and Insulin Fasting and Postprandial Contents

After taking 75 g glucose solutions, blood glucose levels significantly differed at 30, 60, 90, 120, and 180 min, as compared with 0 min (*p* < 0.001) (Table 6). After taking 75 g glucose solutions with djulis hull powder (meals), the blood glucose level after 120 min of eating was still significantly higher than the fasting blood glucose level. It took 120–180 min for the blood glucose level to gradually decrease to the fasting blood sugar level. Eating 10 g or 5 g djulis hull powder before meals did not affect the postprandial blood glucose recovery time. In addition, it took 120–180 min to return to the fasting blood sugar level.

Table 7 shows the postprandial blood insulin content changes for patients. After consuming 75 g glucose, with 10 g djulis hull powder, the insulin content significantly differed at 30, 60, 90, 120, and 180 min, as compared with 0 min after the meal (*p* < 0.05). Insulin content significant differed at 60 and 120 min from 0 min (*p* < 0.05). Yet, after eating 5 g djulis hull powder and 75 g glucose, the insulin content at only 60 and 180 min after the meal significantly differed from 0 min (*p* < 0.05). The results clarified that the change in postprandial insulin content differed from the change in blood glucose level, gradually increased with increasing postprandial time, and then decreased. This result may be related to the inclusion of patients with differing pancreatic and metabolic metabolism rates.

#### 3.3.3. Related Factors Affecting Postprandial Blood Glucose and Insulin Contents

The different gender of patients with different BMIs may affect the response of blood glucose and insulin. Therefore, we used a linear-regression model to analyse the factors affecting postprandial blood glucose and insulin contents by gender. Gender, age, DM history, and BMI were not significantly associated with postprandial blood glucose and insulin concentrations (Table 8). Thus, the results were not discussed separately by gender. The linear-regression model produced with GEE method revealed that postprandial blood glucose level was positively associated (*p* < 0.001) with the postprandial times of 30, 60, 90, and 120 min. With increasing postprandial time, the postprandial blood glucose increased gradually, and from 120 min it tended to slow; postprandial blood glucose and fasting blood glucose did not reach a significant difference until 180 min.

We tested for haemoglobin A1C (HbA1c; glycated haemoglobin or glycohemoglobin. A1C and blood glucose contents showed a significant positive relation (*p* < 0.001). Moreover, for each 1% increase in A1C, blood glucose level increased by 51.4 mg/dL. Chau et al. [37] clarified that the insoluble fibre-rich fraction, as well as the IDF, AISs, and WISs play an important role in adsorbing glucose, retarding glucose diffusion, and inhibiting α-amylase activity to different extents.

The effects of different doses of djulis hull on postprandial blood glucose were not the same. Consuming 10 g djulis hull produced a negative correlation with blood glucose levels. Consuming 10 g djulis hull reduced blood sugar levels after a meal but not significantly. Because of only 6 patients who consumed 10 g, more data are needed to determine its impact on blood glucose. Consuming 5 g djulis hull produced a positive relation with blood glucose content (*p* < 0.05). Consuming 5 g of djulis hull increased postprandial blood glucose content, probably because the amount of dietary fibre contained in djulis hull can only delay the increase in postprandial blood glucose content and shift the peak of postprandial blood glucose content backward, but is not sufficient to lower postprandial blood glucose content. In addition, the blood glucose value was collected at 180 min after a meal in this study, so the blood glucose value still did not completely decrease. 

Gender, age, DM history, BMI, and different amounts of djulis hull consumed (10 g or 5 g) were no related to postprandial insulin content. Nevertheless, consumption at 30, 60, 90, 120, and 180 min after meals was significantly positively related (*p* < 0.05) to insulin content. This result is consistent with a significant positive correlation between postprandial time and blood glucose content. After adjusting for gender, age, DM history, BMI, and body fat rate, we found a significant relation between postprandial insulin content and postprandial time. Still, we found no significant difference in blood glucose values between 180 min after a meal and at 0 min but did find a significant positive relation for insulin content at 180 min after a meal. Insulin secretion of the diabetic patient may be slow, and the insulin concentration may still be high at 180 min after a meal. Body fat (%) was not significantly related to blood glucose content but was significantly positively related to insulin content (*p* < 0.001). Because body fat tissue is related to the development of insulin resistance, a high body fat ratio may be associated with insulin resistance, resulting in increased secretion [38].

#### 3.3.4. Effect of Djulis Hull on Postprandial Blood Glucose Response Curve

On consuming 10 g djulis hull before 75 g glucose solution, the blood glucose values at 30 and 60 min after the meal were significantly lower than at the same postprandial time without consuming djulis hull (245.5 ± 34.4 vs 167.0 ± 46.7 mg/dL, *p* < 0.05; 297.7 ± 55.9 vs 206.5 ± 56.8 mg/dL, *p* < 0.05) (Table 9). After consuming 5 g djulis hull, blood glucose values did not differ at 0, 30, 60, 90, 120, 180 min from those who did not consume it. After investigating the postprandial blood glucose response graphs for 10 patients and comparing the consumption of hypoglycemic drugs, the effect of djulis hull on postprandial blood glucose may be related to the pancreas function of patients. For patients who did not take hypoglycemic drugs and have good pancreatic function, 5 g djulis hull consumption could significantly improve the postprandial blood glucose content. Furthermore, patients taking only biguanide oral hypoglycemic drugs and 10 g djulis hull consumption could also enhance their postprandial blood glucose response. For those with poor pancreatic function, with insulin secretagogues or insulin, consuming 10 g djulis hull before meals may only delay the increase in blood sugar, and the postprandial blood glucose response curve will be delayed, but the consumption will not affect the postprandial blood glucose response. Data from more patients and adding the pancreatic function test is needed to further confirm the interrelationship between each variable.

## 4. Conclusions

The hull of djulis, which is usually considered to be agricultural waste, is disposed of in landfills and causes some environmental problems. In recent years, many studies have investigated the functional properties of djulis hull. We found that it is a good source of valuable ingredients that contain a high amount of dietary fibre. Furthermore, for patients with T2DM, consuming djulis hull 30 and 60 min before a meal significantly reduced blood glucose content as compared with patients at the same postprandial times who did not consume it. Nevertheless, more participants or patients need to be involved in further study to prevent limitations. The use of djulis hull may increase in the coming years. Consequently, the use of it as a value-added product, particularly for T2DM, may help decrease biomass waste.

## Figures and Tables

**Table 1:** Types of dietary fibre content of djulis hull powder (% dry wt).

AISs	WISs	Dietary Fiber		
		IDF	SDF	TDF
**84.27 ± 0.67**	81.17 ± 0.33	71.54 ± 0.27	3.72 ± 0.45	75.21 ± 0.17

AISs, alcohol insoluble solid; WISs, water insoluble solid; IDF, insoluble dietary fiber; SDF, soluble dietary fiber; TDF, total dietary fiber. Data are mean ± SD of triplicate experiments.

**Table 2:** Physicochemical properties of types of dietary fibre from djulis hull. Different letters indicate differences between values.

Figure	Bulk Density (g/mL)	Water-Holding Capacity (mL/g)	Swelling Property (mL/g)	Oil-Holding Capacity (g/g)
Cellulose	0.29 ^c^ ± 0.05	3.01 ^c^ ± 0.19	7.01 ^b^ ± 0.22	2.77 ^c^ ± 0.09
IDF	0.33 ^bc^ ± 0.11	5.27 ^a^ ± 0.13	7.78 ^a^ ± 0.24	3.16 ^b^ ± 0.17
AISs	0.54 ^a^ ± 0.14	4.02 ^b^ ± 0.17	5.72 ^d^ ± 0.31	3.60 ^a^ ± 0.05
WISs	0.37 ^b^ ± 0.09	4.52 ^b^ ± 0.24	6.69 ^c^ ± 0.17	3.49 ^ab^ ± 0.11

**Table 3:** Effect of djulis hull dietary fibre on glucose diffusion rate. Different letters indicate differences between values.

Fibers	Glucose in Dialysate (µM)				
	20 min	30 min	60 min	120 min	180 min
Control	126 ^a^ ± 1.86	203 ^a^ ± 3.72	463 ^a^ ± 2.48	736 ^a^ ± 1.24	866 ^a^ ± 3.10
Cellulose	114 ^b^ ± 0.62	181 ^b^ ± 1.24	440 ^b^ ± 1.86	689 ^b^ ± 2.48	815 ^b^ ± 1.86
IDF	110 ^c^ ± 1.24	172 ^c^ ± 0.62	411 ^c^ ± 1.24	684 ^b^ ± 1.86	805 ^c^ ± 4.96
AISs	100 ^d^ ± 0.62	167 ^d^ ± 3.10	399 ^d^ ± 0.62	657 ^d^ ± 4.96	794 ^d^ ± 2.48
WISs	108 ^c^ ± 1.86	168 ^d^ ± 1.86	394 ^d^ ± 4.34	675 ^c^ ± 0.62	801 ^c^ ± 0.62

**Table 4:** Effect of insoluble dietary fibre from djulis hull on α-amylase activity. Different letters indicate differences between values.

Fibers	Glucose Produced (µM/h)
Control	7.85 ^a^ ± 0.24
Cellulose	5.96 ^c^ ± 0.14
IDF	6.01 ^b^ ± 0.66
AISs	2.07 ^d^ ± 0.04
WISs	2.47 ^d^ ± 0.22

**Table 5:** Basic information for participants with T2DM.

Basic	Djulis Hull 10 g (*n* = 6)		Djulis Hull 5 g (*n* = 4)		*p*-Value
Information	Mean ± SD	Range	Mean ± SD	Range	
DM history (year)	5.9 ± 5.1	0.3–15.0	3.3 ± 1.7	1.0–4.0	0.604
Age (years)	51.2 ± 14.9	32–71	64.3 ± 11.1	50–73	0.174
Height (cm)	156.5 ± 7.5	147.0–67.0	167.0 ± 7.9	159.0–176.0	0.087
Body weight (kg)	65.3 ± 11.1	52.8–81.7	76.7 ± 14.8	56.1–90.7	0.201
BMI (kg/m^2^)	26.5 ± 2.5	22.6–30.4	27.3 ± 3.4	22.2–29.3	0.522
Muscle weight (kg)	41.0 ± 7.9	34.0–53.2	50.9 ± 9.7	39.0–62.6	0.088
Fat weight (kg)	21.8 ± 3.4	16.7–25.4	22.9 ± 5.7	14.8–28.1	0.670
Body fat (%)	33.6 ± 2.9	31.1–38.1	29.7 ± 3.6	26.3–33.9	0.136
Waist/hip ratio	0.9 ± 0.0	0.9–1.0	1.0 ± 0.1	0.9–1.0	0.516
A1C (%)	6.9 ± 1.0	5.9–8.5	6.1 ± 0.5	5.7–6.8	0.165
Fasting glucose (mg/dL)	133.5 ± 40.0	205.0–80.0	129.5 ± 15.6	149.0–116.0	0.831
Insulin (mg/dL)	6.8 ± 6.3	18.4–0.1	9.4 ± 4.4	14.8–5.1	0.522

*p*-value by Mann-Whitney U Test; A1C, haemoglobin A1C.

**Table 6:** Postprandial blood glucose levels in patients with T2DM after 75 g glucose and djulis hull consumption.

	Djulis Hull 10 g (*n* = 6)		Djulis Hull 5 g (*n* = 4)		Overall	
	Mean ± SD	*p*-Value	Mean ± SD	*p*-Value	Mean ± SD	*p*-Value
**No consumption of djulis**						
0 min	133.5 ± 40.7		129.5 ± 15.5		131.9 ± 31.7	
30 min	245.5 ± 34.4	<0.001 **	213.8 ± 26.1	<0.001 **	232.8 ± 34.0	<0.001 **
60 min	297.7 ± 55.9	<0.001 **	262.5 ± 27.1	<0.001 **	283.6 ± 48.1	<0.001 **
90 min	297.3 ± 74.3	<0.001 **	276.3 ± 28.7	<0.001 **	288.9 ± 58.8	<0.001 **
120 min	250.7 ± 86.7	<0.001 **	244.0 ± 47.0	<0.001 **	248.0 ± 70.1	<0.001 **
180 min	155.2 ± 81.0	0.335	125.5 ± 49.1	0.787	143.3 ± 68.4	0.455
**Consumption of Djulis**						
0 min	140.3 ± 46.1		124.5 ± 22.5		134.0 ± 37.7	
30 min	167.0 ± 46.7	0.001 *	178.3 ± 43.5	<0.001 **	171.5 ± 43.3	<0.001 **
60 min	206.6 ± 56.8	<0.001 **	236.8 ± 60.4	<0.001 **	218.6 ± 57.0	<0.001 **
90 min	238.8 ± 79.8	<0.001 **	251.5 ± 55.8	<0.001 **	243.9 ± 68.0	<0.001 **
120 min	243.0 ± 92.6	0.001 *	226.8 ± 72.0	<0.001 **	236.5 ± 81.0	<0.001 **
180 min	183.7 ± 108.0	0.123	171.8 ± 76.4	0.098	178.9 ± 92.0	0.027 *

Results of linear-regression model with generalized estimating equations; * *p* < 0.05 compared to 0 min; ** *p* < 0.01 compared to 0 min.

**Table 7:** Postprandial insulin concentration levels in patients with T2DM after 75 g glucose and djulis hull consumption.

	Djulis Hull 10 g (*n* = 6)		Djulis Hull 5 g (*n* = 4)		Overall	
	Mean ± SD	*p*-Value	Mean ± SD	*p*-Value	Mean ± SD	*p*-Value
**Not consumption of djulis**						
0 min	6.8 ± 6.3		9.4 ± 4.4		7.8 ± 5.5	
30 min	15.7 ± 10.3	0.003 *	26.0 ± 28.2	0.113	19.8 ± 18.8	0.019 *
60 min	33.7 ± 16.6	0.012 *	58.1 ± 56.2	0.032 *	43.5 ± 36.9	<0.001 **
90 min	48.1 ± 28.8	<0.001 **	108.7 ± 130.1	0.069	72.3 ± 84.2	0.008
120 min	26.0 ± 16.7	<0.001 **	121.7 ± 165.8	0.019 *	64.3 ± 108.4	0.076
180 min	23.4 ± 20.2	0.044 *	28.0 ± 29.2	0.086	25.2 ± 22.7	0.002 *
**Consumption of Djulis**						
0 min	28.2 ± 52.4		11.0 ± 4.8		21.3 ± 40.3	
30 min	29.2 ± 56.2	0.431	16.2 ± 16.9	0.441	24.3 ± 43.6	0.319
60 min	39.6 ± 54.0	0.100	41.5 ± 24.9	0.001 *	40.3 ± 42.7	0.030 *
90 min	47.3 ± 52.8	0.063	21.5 ± 35.4	0.443	37.0 ± 46.3	0.061
120 min	55.5 ± 57.2	0.025 *	25.1 ± 22.5	0.101	43.3 ± 47.3	0.008 *
180 min	26.2 ± 45.6	0.529	33.1 ± 24.8	0.038 *	29.0 ± 37.0	0.200

Results of linear regression model with generalized estimating equations; * *p* < 0.05 compared to 0 min. ** *p* < 0.01 compared to 0 min.

**Table 8:** Regression analysis of factors associated with postprandial blood glucose and insulin content.

		Blood Glucose (mg/dL)				Insulin (mg/dL)			
Parameter		Estimate	SE	95% C.I.	*p*-Value	Estimate	SE	95% C.I.	*p*-Value
**Sex**	**Male**	−6.6	22.2	−50.1; 37.0	0.768	15.0	14.9	−14.2; 44.3	0.314
	**Female**	0.0				0.0			
**Age**		−0.27	0.5	−1.3; 0.7	0.584	0.4	0.48	−0.9; 1.0	0.928
**Diabetes history (years)**		0.97	1.0	−0.9; 2.9	0.31	0.07	1.06	−2.0; 2.1	0.947
**Djulis hull, g**	**10**	−4.2	15.6	−34.7; 26.3	0.788	9.2	19.0	−28.0; 46.4	0.627
	**5**	19.6	9.6	0.7; 38.5	0.042 *	−6.2	12.8	−31.4; 18.9	0.628
**After meal, min**	**180**	21.2	14.9	−7.9; 50.4	0.153	9.9	2.6	4.8; 15.0	<0.001 **
	**120**	103.4	14.0	75.9; 131.0	<0.001 **	37.3	16.2	5.6; 69.1	0.021 *
	**90**	128.7	9.9	109.4; 148.0	<0.001 **	38.8	14.6	10.1; 67.4	0.008 *
	**60**	114.6	7.3	100.2; 129.0	<0.001 **	26.5	6.6	13.5; 39.5	<0.001 **
	**30**	67.2	5.4	56.7; 77.7	<0.001 **	7.1	2.5	2.3; 11.9	0.004 *
	**0**	0.0				0.0			
**A** **1C (%)**		51.4	6.1	39.5; 63.3	<0.001 **	−16.1	7.8	−31.4; 0.9	0.038 *
**Body fat (%)**		−1.6	2.1	−5.8; 2.6	0.454	4.8	1.1	2.7; 7.03	<0.001 **
**BMI, kg/m^2^**		−3.3	2.6	−8.5; 1.8	0.208	4.1	2.8	−1.4; 9.5	0.142

Results of linear regression model with generalized estimating equations. 95% CI confidence interval; BMI, body mass index; A1C, haemoglobin A1C. * *p* < 0.05 compared to 0 min; ** *p* < 0.01 compared to 0 min.

**Table 9:** Effect of djulis hull consumption on postprandial blood glucose content (mg/dL). The asterisk indicates the significance of the value.

		Djulis Hull 10 g (*n* = 6)		Djulis Hull 5 g (*n* = 4)	
		Mean ± SD	*p*-Value	Mean ± SD	*p*-Value
0 min	No consumption	133.5 ± 40.7	0.116	129.5 ± 15.5	0.273
	Consumption	140.3 ± 46.1		124.5 ± 22.5	
30 min	No consumption	245.5 ± 34.4	0.028 *	213.8 ± 26.1	0.068
	Consumption	167.0 ± 46.7		178.3 ± 43.5	
60 min	No consumption	297.7 ± 55.9	0.027 *	262.5 ± 27.1	0.465
	Consumption	206.5 ± 56.8		236.8 ± 60.4	
90 min	No consumption	297.3 ± 74.3	0.116	276.3 ± 28.7	0.461
	Consumption	238.8 ± 79.8		251.5 ± 55.8	
120 min	No consumption	250.7 ± 86.7	0.753	244.0 ± 47.0	0.715
	Consumption	243.0 ± 92.6		226.8 ± 72.0	
180 min	No consumption	155.2 ± 81.0	0.345	125.5 ± 49.1	0.068
	Consumption	183.7 ± 108.0		171.8 ± 76.4	

## Data Availability

MDPI Research Data Policies at https://www.mdpi.com/ethics (accessed on 5 February 2021).
